# Characterization of stem/progenitor cell cycle using murine circumvallate papilla taste bud organoid

**DOI:** 10.1038/srep17185

**Published:** 2015-11-24

**Authors:** Eitaro Aihara, Maxime M. Mahe, Michael A. Schumacher, Andrea L. Matthis, Rui Feng, Wenwen Ren, Taeko K. Noah, Toru Matsu-ura, Sean R. Moore, Christian I. Hong, Yana Zavros, Scott Herness, Noah F. Shroyer, Ken Iwatsuki, Peihua Jiang, Michael A. Helmrath, Marshall H. Montrose

**Affiliations:** 1 Department of Molecular and Cellular Physiology, University of Cincinnati, Cincinnati, Ohio; 2 Division of Pediatric Surgery, Cincinnati Children’s Hospital Medical Research Center, Cincinnati, Ohio; 3 Monell Chemical Senses Center, Philadelphia, Pennsylvania; 4 Division of Gastroenterology, Hepatology, and Nutrition, Cincinnati Children’s Hospital Medical Research Center, Cincinnati, Ohio; 5 Division of Oral Biology, The Ohio State University, Columbus, Ohio; 6 Department of Nutritional Science and Food Safety, Tokyo University of Agriculture, Tokyo, Japan

## Abstract

Leucine-rich repeat-containing G-protein coupled receptor 5-expressing (Lgr5^+^) cells have been identified as stem/progenitor cells in the circumvallate papillae, and single cultured Lgr5^+^ cells give rise to taste cells. Here we use circumvallate papilla tissue to establish a three-dimensional culture system (taste bud organoids) that develops phenotypic characteristics similar to native tissue, including a multilayered epithelium containing stem/progenitor in the outer layers and taste cells in the inner layers. Furthermore, characterization of the cell cycle of the taste bud progenitor niche reveals striking dynamics of taste bud development and regeneration. Using this taste bud organoid culture system and FUCCI2 transgenic mice, we identify the stem/progenitor cells have at least 5 distinct cell cycle populations by tracking within 24-hour synchronized oscillations of proliferation. Additionally, we demonstrate that stem/progenitor cells have motility to form taste bud organoids. Taste bud organoids provides a system for elucidating mechanisms of taste signaling, disease modeling, and taste tissue regeneration.

The five basic taste qualities (sweet, sour, salty, bitter, and umami) are sensed by taste receptor cells within the taste buds of the tongue^1^,^2^. Primary taste culture has been attempted to model the function of taste cells with variable degrees of success^3^,^4^. However, because taste cells are terminally differentiated and have limited lifespan, use of primary cultures has not been amenable to studies of development and differentiation^5^,^6^,^7^. Studies of proliferation and pulse-chase experiments suggested that stem/progenitor cells surround the base of taste buds^8^,^9^,^10^. Recent reports demonstrate Leucine-rich repeat-containing G protein-coupled receptor 5 positive (Lgr5^+^) stem cells are present at the trench area and the base of the taste buds in circumvallate (CV) papilla tissue^11^,^12^.

Based on recent advances in understanding of stem cell biology in the gastrointestinal tract epithelium, a novel long-term primary culture method has been developed whereby three-dimensional (3D) structures called organoids are generated from Lgr5^+^ stem cells isolated from the mouse or human small intestinal crypt base^13^,^14^. This approach has been extended to stomach^15^, colon^16^, liver^17^, and pancreas^18^. Importantly, these tissue-derived organoids can stably express differentiated cell types specific to the native organ. These gastrointestinal organoids consist of a simple epithelial cell monolayer in which cells are connected by apically oriented tight junctions. More recently, Lgr5^+^ sorted single stem cells from the circumvallate papillae have been shown to successfully generate organoids containing differentiated taste cells^19^, however primary culture of tissue-derived taste bud organoids has not been established.

The cell cycle duration of stem/progenitor cells in the native tissue are mostly determined by endpoint quantitative analysis through detecting proliferative or mitotic cells in the fixed tissue section. Since this method is static and not a real-time analysis, it cannot detect all populations of the proliferative cell cycle. Nevertheless, several studies in the small intestine have suggested that the Lgr5^+^ stem cell cycle is approximately 24 hours^20^,^21^, while cell cycle estimates for the transient amplifying zone are approximately 12 hours^22^,^23^. Interestingly, in the taste bud proliferative cells, there are several cell cycle populations calculated by labeling proliferative cells^10^. To determine the cell cycle in real-time of these distinct populations, we employed the FUCCI2 system in which mCherry-hCdt1 (30/120) (red fluorescence) is expressed during G1 phase while mVenus-hGem (1/110) (green fluorescence) is expressed during the S/G2/M phase of the cell cycle^24^.

Herein we demonstrate successful development of taste bud organoids derived from native CV tissue. The taste bud organoid has phenotypic characteristics similar to native taste tissue, including a multilayered epithelium containing stem/progenitor in the outer layers and differentiated epithelial taste cells in the inner layers. Our data indicate that stem/progenitor cells have distinct cell cycles marking five separable populations of cells. Furthermore we demonstrate that proliferative cells do not sustain a single fixed position in the organoid. This suggests that stem/progenitor cells can reposition within the circumvallate papilla and contribute to the maintenance of taste tissue during homeostatic turnover of cells and regeneration *in vivo*. Thus, the taste bud organoid model is a uniquely beneficial tool to investigate stem/progenitor function in this tissue.

## Results

### Generation of taste bud organoids from circumvallate tissue

**Figure 1:**
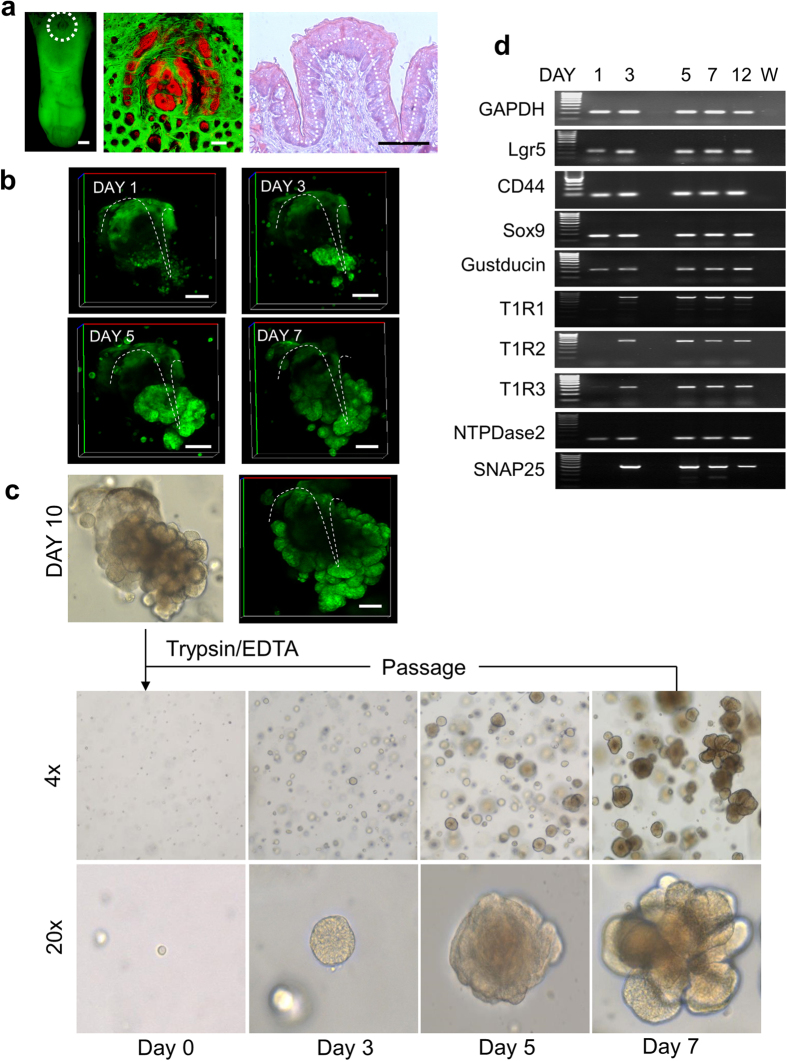
Development of taste bud organoids from CV tissue. (**a**) Isolated tongue from YC mouse (*white circle area*: circumvallate papilla, GFP filter, Scale bars = 1000 μm), confocal image and H&E staining of CV (*white dotted line*: epithelium, Scale bars = 200 μm). Nuclei stained with Hoechst 33342 (Red). **(b)** 3D structure of taste bud organoid grows from isolated CV (*white circle area* in (**a**) from YC mouse. 3D YFP reconstructed from 1, 3, 5 or 7 day cultures. The *white dotted line* shows morphology of isolated CV epithelium. Scale bars = 100 μm **(c)** After 10 days in culture, taste bud organoids were broken up to single cells by trypsin/EDTA and re-embedded in Matrigel. Images were taken 0, 3, 5 or 7 days after passage. **(d)** Stem/progenitor cell or taste bud lineage marker mRNA was determined 1, 3, 5, 7, or 12 days after passage. W: water.

Isolated CV tissue (Fig. 1a) was embedded in Matrigel and overlaid with growth medium adapted for taste bud tissue (described in Methods) to generate taste bud organoids (Fig. 1b). Yellow fluorescent protein (YFP)-expressing mice (Yellow cameleon transgenic: YC mice)^25^ were used to generate cultures. We observed organoid generation from the downward facing side of isolated CV tissue suggesting progenitor cells reside at the base of this tissue (Fig. 1b). In contrast, isolated epithelial tissues adjacent to CV failed to generate organoid structures (Supplementary Fig. 1 “region 2 and 3”). At culture Day 10 from CV tissue, organoids were digested to single cells using 0.25% trypsin/EDTA, passed through 31 G insulin syringe (10^3^ cells) and re-embedded to Matrigel (1 passage). Successive digestions and re-embedding of single cells resulted in an increased number of organoids, suggesting that these culture methods select for and expand stem/progenitor cell populations during organoid growth (Fig. 1c and Supplementary video 1 and 2). To determine if taste bud organoids express markers of native tissue, we performed RT-PCR analysis and detected the presence of Lgr5 (stem/progenitor cell markers), CD44 (stem/progenitor cell markers) and Sox9 (progenitor markers) in organoids that have been sequentially passaged at least 2 times (Fig. 1d). Furthermore, after passage, the taste bud organoids re-expressed all of the major taste cells markers: e.g. TYPE I cell marker: nucleoside triphosphate diphosphohydrolase-2 (NTPDase2), TYPE II cell markers: T1R1,2,3 and gustducin, TYPE III marker: SNAP25 (Fig. 1d). We also detected the presence of mRNA for calcium channels that have been implicated in taste signaling, including TRPV1 and TRPM5, and the calcium sensing receptor (CaSR) in the taste bud organoid (Supplementary Fig. 2a).

To determine the optimal growth conditions for taste bud organoids, we performed trials of growth factor removal followed by measuring growth efficiency, ability to form buddings and expression of mature taste cells. EGF has been identified as an essential component of growth media for organoid generation^26^, and our results demonstrate its presence is also required for growth of taste bud organoids (Supplementary Fig. 3). Other required growth factors that have been shown to maintain stem cell activity in the organoid culture system are Noggin (a BMP inhibitor) and R-spondin and Wnt3a (drivers of Lgr5 signaling). When Noggin or R-spondin was removed from media, taste bud organoids formed, however digestion and re-embedding of organoids was not successful (Supplementary Fig. 3a and 3b). Likewise, without Wnt3a, organoids lost the ability to form buddings and passage efficiency was dramatically decreased (Supplementary Fig. 3a and 3b). Gene expression was compared among organoids grown under different culture conditions, as well as compared to small intestinal organoid culture and native CV tissue. Use of all growth factors in the taste bud organoid resulted in a gene expression pattern mirroring that of native CV tissue (Supplementary Fig. 3c). Interestingly, we failed to detect taste receptor genes, T1R2 and SNAP25, in the Wnt3a removal condition (Supplementary Fig. 3c). These results suggested that EGF, Wnt3a, R-spondin and Noggin play important roles in the maintenance of stem cell activity and organoid formation, and Wnt signaling is involved in the differentiation to mature taste cells.

Taste and gut tissues share similarity in many aspects, including signaling and development. Therefore, we further compared mRNA expression patterns of taste bud organoids with gastrointestinal (GI) organoids cultured with organoid medium conditions. In Supplementary Fig. 2 and 3c, all the examined taste cell related mRNA expression was present in the taste bud organoids, whereas some taste cells markers, such as T1R2 mRNA were not detected in GI organoids. Interestingly, among the GI organoids, antral gastric organoids expressed the most taste cell-related genes (Supplementary Fig. 2). Together, these finding identify a regional pattern of gene expression specificity in CV tissue that is shared with other endoderm organs.

### Expression of taste cells and stem/progenitor cells in the taste bud organoid

**Figure 2:**
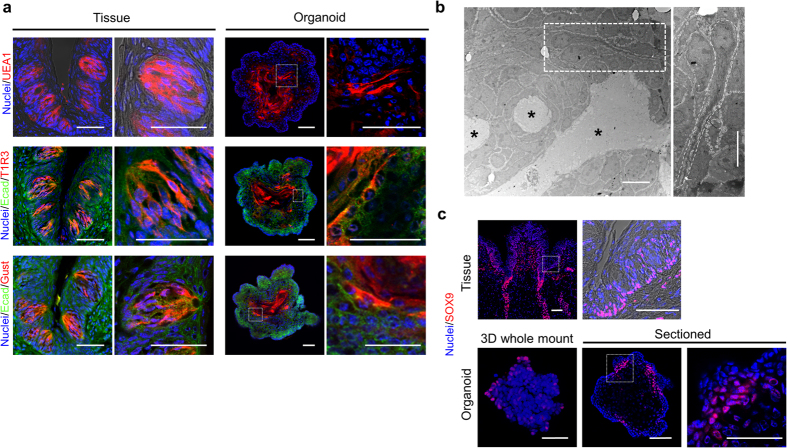
Expression of taste bud cell lineage markers in the organoid. (**a**) Sectioned CV tissue or organoid were stained with UEA1 (red), E-cadherin (green) and T1R3 (red) or E-cadherin (green) and gustducin (red), respectively. Cell nucleus (blue) was stained by Hoechst 33342. Scale bars = 50 μm. (**b**) Transmission electron microscopy images shows the middle of the organoid with low resolution, and high resolutions of taste cell (*white rectangle*). Asterisks indicate the lumen. Scale bars = 10 μm. (**c**) Sectioned CV tissue (low magnification and high magnification of *white rectangle*), 3D whole mount organoid, or sectioned organoid (low magnification and high magnification of *white rectangle*) were stained with SOX9 (red). Nuclei (blue) was stained with Hoechst 33342. Scale bars = 50 μm.

Consistent with previous observations^27^, *Ulex europeaus agglutinin*-I (UEA1) labelled intragemmal taste bud cells (Fig. 2a**: Tissue)**. Furthermore, taste cell markers T1R3 and gustducin were specifically detected in taste bud cells in CV tissue (Fig. 2a**: Tissue)**. We observed that UEA1 positive cells were localized inside the taste bud organoids (Fig. 2a**: Organoid)**. Likewise, T1R3 and gustducin positive taste cells were found in the inner layer of the organoid (Fig. 2a**: Organoid)**. In electron microscopy, we further observed the existence of presumably taste-like cells showing a 5 μm soma, and 40–50 μm length with a basally located nucleus (Fig. 2b). We observed the existence of a lumen in the middle of the organoids (Fig. 2b, ***asterisk***, and Supplementary Fig 4). In contrast, SOX9, the marker for stem/progenitor cells^28^,^29^, was strongly expressed in the trench and weakly expressed in the base of the taste buds of CV (Fig. 2c**: Tissue)**. Interestingly, SOX9 was expressed in the outer layer of the organoid, confined mainly to budding regions (Fig. 2c**: Organoid)**. These data suggest that differentiated taste cells are located within the inner cell layer of taste bud organoids while stem/progenitor cells are present within the outer cell layer.

To confirm that the taste bud organoid growth patterns occur within multiple layers, we generated organoids from H2B-EGFP, or membrane-tdTomato transgenic mice. In contrast to other gastrointestinal-derived organoids that grow in three dimensions with a simple epithelial monolayer, Supplementary Fig. 5 shows multiple cell layers in the taste bud organoid, consistent with growth *in vivo*.

### Taste bud organoids can be generated from Lgr5^+^ or CD44^+^ stem/progenitor cells

**Figure 3:**
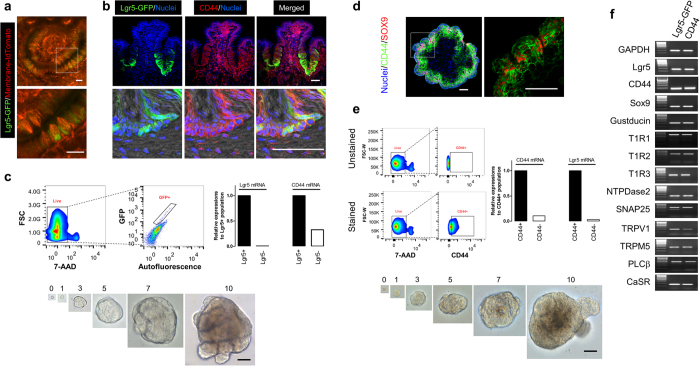
Lgr5^+^ or CD44^+^ cell-induced taste bud organoids. (**a**) 3D images CV region of tongue freshly isolated from Lgr5-GFP/membrane-tdTomato mouse with low magnification and high magnification of *white rectangle* indicated in top panel. Scale bars = 50 μm. (**b**) Sectioned Lgr5-GFP mouse CV tissue was immunostained for GFP (green), CD44 (red) and nuclei (Hoechst 33342: blue). Scale bars = 50 μm. (**c**) Representative histograms of Lgr5-GFP stem cell sorting from isolated CV tissue, and expression of Lgr5 or CD44 mRNA were determined. Images of taste bud organoid growth from sorted Lgr5-GFP stem cell, at indicated days after cell plating. (**d**) Sectioned organoid was stained with CD44 (green), SOX9 (red) and nuclei (blue). Scale bars = 50 μm. (**e**) Representative histograms of CD44^+^ cell sorting from taste bud organoids, and expression of Lgr5 or CD44 mRNA were determined. Images of taste bud organoid growth from sorted CD44^+^ cell. (**f**) After 12 days culture of Lgr5-GFP cell or CD44^+^ cell-derived taste bud organoid, stem/progenitor cell or taste bud lineage markers mRNA was determined by RT-PCR.

Similar to native tissue, we detected Lgr5 mRNA in the taste bud organoid. It was recently reported that sorted Lgr5-GFP single cells successfully generated taste bud organoids^19^. Here we confirmed that Lgr5-GFP cells were found in the base of taste buds and CV (Fig. 3a,b), and single Lgr5-GFP cells formed taste bud organoids (Fig. 3c), consistent with previous studies^11^,^19^. However, as described previously, the appearance of Lgr5-GFP cells within the taste bud organoids is rare^19^, therefore we asked if CD44 can substitute as a broad stem/progenitor cells marker^30^,^31^ in the taste bud organoid. We observed that CD44 is expressed in both taste bud cells and cells at the base of taste buds in CV, and co-localized with Lgr5-GFP stem/progenitor cells (Fig. 3b). We detected high CD44 expression in the sorted Lgr5-GFP single cells (Fig. 3c). In contrast, in the taste bud organoid derived from CV tissue, CD44 was found more extensively in the outer layer of the taste bud organoids where SOX9 was expressed (Fig. 3d), suggesting that cells arise from progenitor/stem cells in the outer layer and differentiate toward the inner layer to form adult taste bud organoids. To confirm this hypothesis, we asked if sorted CD44^+^ single cells from taste bud organoids were able to form taste bud organoids. As expected, culture of these cells resulted in the formation of taste bud organoids (Fig. 3e). Both Lgr5^+^ and CD44^+^ cell-derived organoids expressed all taste cell lineage mRNAs (Fig. 3f). It is noted that high Lgr5 expression was detected in the sorted CD44 single cells from taste bud organoids (Fig. 3e), suggesting these markers are co-expressed throughout the organoid.

### Proliferative zone in taste bud organoids

**Figure 4:**
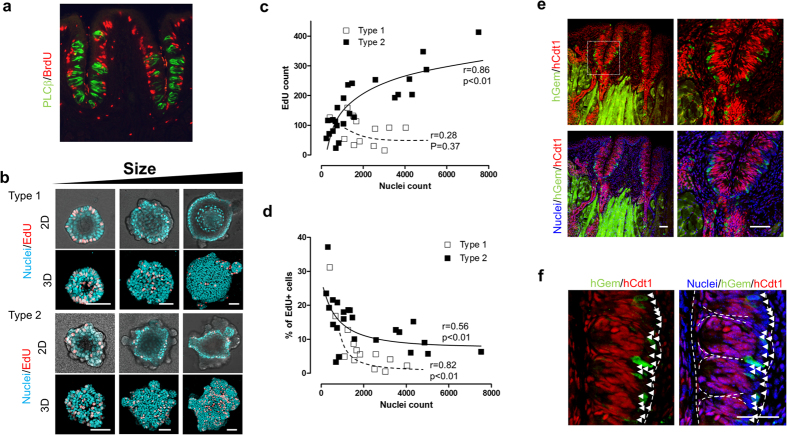
Proliferation zone within taste bud organoids. Representative images of BrdU (red) staining in the CV tissue (PLCβ:green) (**a**), 2D (top) and 3D (bottom) EdU (red) staining of different sizes of type 1 (sphere) or type 2 (with budding) taste bud organoid (**b**). (**c**) Numbers of EdU positive cells were counted in the different sizes of type 1 (sphere: open rectangle) or type 2 (with budding: closed rectangle) taste bud organoid. **(d)** % EdU positive cells versus nuclei were calculated from different sizes of type 1 (sphere: open rectangle) or type 2 (with budding: closed rectangle) taste bud organoid shown in (**c)**. (**e**) Representative images of expression of mVenus (green) and mCherry (red) in the CV tissue. (**f**) High magnification image of mVenus (green) and mCherry (red) in the CV tissue of *white rectangle* indicated in (**e**). Symbol ▵ shows cells that both mVenus (green) and mCherry (red) did not detect. Nuclei (blue) was stained with Hoechst 33342. The *white dotted line* shows morphology of taste buds. Scale bars = 50 μm (**b**,**e**,**f**).

To confirm our observation that stem/progenitor cells are present in the outer layer of taste bud organoids, we performed a proliferation assay. We found that BrdU (5-bromo-2′-deoxyuridine) positive cells were found along the base of the taste bud in the CV tissue (Fig. 4a), consistent with others findings^10^,^32^. Furthermore we found that proliferation occurred only in the outer layer of the taste bud organoids as shown by staining of EdU (5-ethynyl-2′-deoxyuridine), a novel derivative of BrdU (Fig. 4b), and the percentage of EdU positive cells among organoids varied with organoid size and length of time in culture (Fig. 4b–d). We identified two morphological types of organoids: 1) a sphere with smaller size buddings that are fewer in number with growth in both sphere and budding areas and 2) a spherical structure in the center with more buddings where most of the growth is in the prominent budding areas (Fig. 4b and Supplementary Fig. 5). Both types of organoids had multiple cell layers and expressed taste cells shown by UEA1 staining in the inner cell layer facing the lumen (Supplementary Fig. 5b). The number of EdU positive cells in type 2 organoids increased in a size-dependent manner, while those in type 1 organoids decreased (Fig. 4c). However, the total percentage of EdU positive cells dramatically decreased when organoids grew to a size greater than 1000 cells (Fig. 4d). These data suggest that stem/progenitor cells are actively proliferating in the budding regions within both types of the taste bud organoid.

To further characterize the pattern of proliferation, we employed FUCCI2 transgenic mouse CV tissue. In the FUCCI2 system, mCherry-hCdt1(30/120) (red fluorescence) is expressed during G1/G0 phase while mVenus-hGem (1/110) (green fluorescence) is expressed during S/G2/M phase of cell cycle^24^,^33^,^34^. Additionally, both green and red fluorescence disappear during cell division (between M and G1 phase)^35^. The cells of resting phase or differentiated cells (G0 phase) sustain mCherry expression in their nuclei^33^,^34^,^35^. In Fig. 4e, we observed that mVenus-hGem positive cells localized at the base of CV tissue, similar to the distribution of BrdU positive proliferative cells. Furthermore, many FUCCI2 negative cells were found at the base of the taste bud (Fig. 4f), suggesting that most cells existing at the base of the taste bud are the stem/progenitor cells. In contrast, taste cells within the taste bud are strongly mCherry-hCdt1 positive, confirming that those are terminally differentiated cells (Fig. 4e,f).

**Figure 5:**
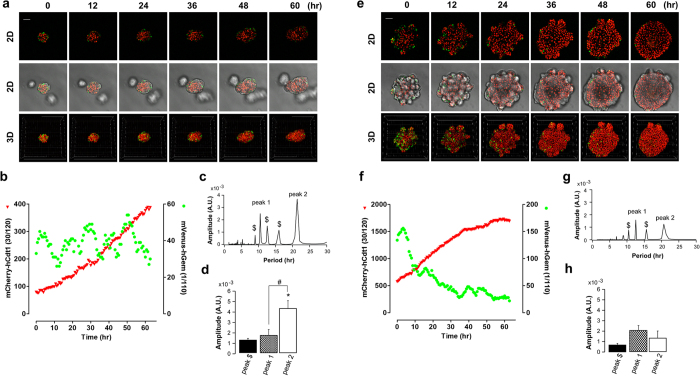
Monitoring the cell cycle using FUCCI2 taste bud organoids. Taste bud organoids were created from FUCCI2 transgenic mice and organoid growth was monitored from 3–6 days **(a)** or 9–12 days **(e)** on the confocal microscope. Scale bars = 50 μm. Numbers of mCherry (red) or mVenus (green) positive cells in 4D were counted using Imaris software **(b** or **f)**. **(c** or **g)** Representative FFT analysis data of time courses of number of mVenus-hGeminin positive cells from organoids monitored from 3–6 days (**a**) or 9–12 days (**e**), respectively. (**d** or **h**) The average amplitudes of peak 1 and peak 2 were calculated from 3 different taste bud organoids. The average amplitude of peak $ was calculated from detected peaks other than peak 1 and peak 2. *p < 0.05 vs. peak $, ^#^p < 0.05 vs. peak 1.

In taste bud organoids created from FUCCI2 transgenic mouse CV tissue, we observed the transient mVenus-hGem fluorescence only within the outer cell layer of organoids, while the inner cell layer expressed only mCherry (Fig. 5). This is consistent with the EdU staining observation in actively proliferating cells, such as stem/progenitor cells, that were present in the outer layer. In the early stage of growth of organoids (3–6 days), the number of mVenus-hGem positive cells presented in rhythmic profile, while mCherry-hCdt1 gradually increased, suggesting that stem/progenitor cells are proliferating in a synchronized manner (Fig. 5a,b and Supplementary video 3a and 3b). We constantly detected peak 1 and peak 2, whereas discrete peaks (peak $) were detected as well. The highest peak numbers of mVenus-hGem positive cells appeared at 21.34 ± 0.01 hours (peak 2) (n = 3) (Fig. 5c), and the amplitude of peak 2 was significantly higher than the secondary peak at 12.45 ± 2.61 hours (peak 1) (Fig. 5d). There was no significant difference between peak $ and peak 1. These suggest that peak 2 is the major peak in the synchronized oscillation of proliferation activity. In contrast, in the late stage of organoid growth (10–12 days), the majority of mVenus-hGem positive cells appeared in the budding region, and decreased when organoids became spheres (with less budding) (Fig. 5e,f and Supplementary video 4a and 4b). We continued to observe peaks at 12.73 ± 2.81 hours (peak 1) and 20.44 ± 0.44 hours (peak 2) (n = 3) consistently (Fig. 5g). However, there was no difference in the amplitude among peaks (Fig. 5h), suggesting the cell proliferation becomes less synchronized in the late phase of growth.

### Cell cycle of stem/progenitor cells

**Figure 6:**
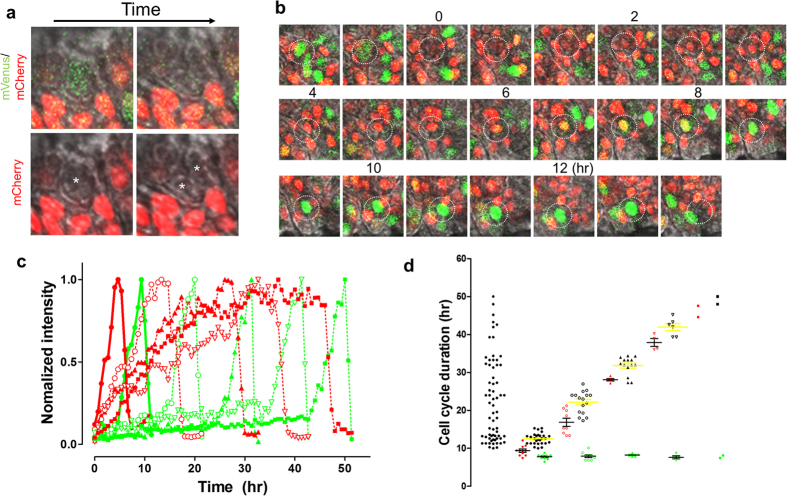
Diversity of cell cycle duration in the taste bud organoid. Taste bud organoids were created from FUCCI2 transgenic mice and growth was monitored by confocal microscope. (**a**) High magnification image of mVenus/mCherry (top), or mCherry (bottom) with differential interference contrast (DIC) superimposed on the fluorescence. Note that cell division (*white asterisk*) occurs following the disappearance of mVenus fluorescence. (**b**) Representative images of 12 hr cell cycle determined by tracking FUCCI2 fluorescence appearance in the taste bud organoid. (**c)** Changes of mVenus or mCherry fluorescence intensity during cell cycle, calculated from **b** (●), Supplementary Fig. **7a** (○), **7b** (▲), **7c** (▽) or from 52 hr cycling cells (■). **(d)** Duration of the cell cycle from 68 cells tracked (left: black circle), and divided into 5 groups based on clustering of cell cycle duration (Supplementary Fig. 6). Each group shows duration of mCherry (red), mVenus (green) (black line) and mCherry + mVenus (black: yellow line).

It has been demonstrated that there are two stem/progenitor cell cycles in the taste bud niche: rapid or slow cycling cells, although only one peak of proliferation (by counting total numbers of BrdU labeled cells) is detected over 24 hours^10^. We observed that the dominant period of mVenus-hGem positive cells occurs about every 24 hours (Fig. 5), suggesting that the proliferation rhythms of taste bud organoids mimic the *in vivo* system. Intriguingly, we also detected additional frequencies, suggesting a heterogeneous cell cycle time. Therefore, we tracked mVenus-mCherry or H2B-EGFP fluorescence to measure cell cycle duration at the single cell level. After mVenus-hGem (S/G2/M) fluorescence disappeared, cell division occurred, followed by the expression of the mCherry-hCdt1 (G1) (Fig. 6a,b) confirming fidelity of the FUCCI2 system for reporting cell cycles in the taste bud organoid. During tracking of individual cells, we found a diversity of several cell cycle durations. The population was divided into 5 categories based on cell cycle duration, 12.8 ± 0.1 (39.4% of total analyzed cells), 22.7 ± 0.3 (26.8%), 30.8 ± 0.3 (24.2%), 41.6 ± 0.6 (7.2%), and 50.3 ± 0.9 hours (2.4%), respectively (Supplementary Fig. 6, Fig. 6c,d, and Supplementary Fig. 7). Interestingly, mVenus-hGem cycle durations were very similar among groups, 5.6 ± 0.2, 5.8 ± 0.4, 6.1 ± 0.2, 5.5 ± 0.4, and 5.7 hours, respectively (Fig. 6d). These results suggest all cells are equally competent to undergo the S/G2/M transitions, but have variable dwelling time in the G1 phase.

**Figure 7:**
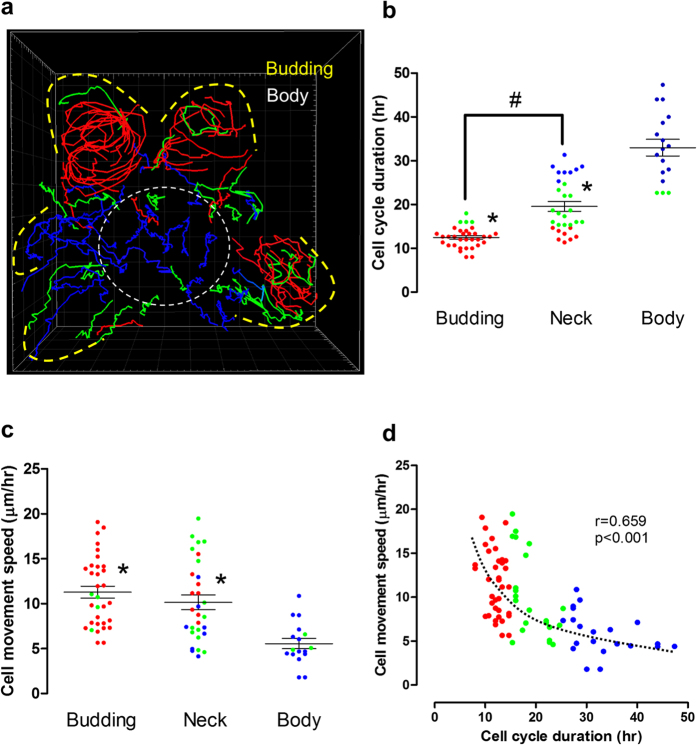
Tracking individual cells in the taste bud organoid. Manual cell tracking was performed using Imaris 7.7 on FUCCI2 organoids showed in Fig. 5e and Supplementary video 4. (**a**) Image showed summary of proliferative cell tracking, while tracking 3D movie showed in Supplementary video 5. The line colors were separated by cell cycle duration (Red: <15 hr, Green: 15–25 hr, Blue >25). The cell cycle durations (**b**) or cell movement speeds (**c**) are shown at different positions in the organoid (In (**a**), Budding: *yellow outlined*, Neck: *between yellow and body*, Sphere Body: *while outlined*). Additionally, correlation between cell movement speed and cell cycle duration was shown in **d**. *p < 0.05 vs. body. ^#^p < 0.05 vs. neck.

We observed that proliferating cells (mVenus-hGem positive) moved along the outer cell layer of the organoid (Fig. 7a and Supplementary video 5). Further, cell cycle times tended to vary according to cell position within the outer layer of the organoid. Cell cycle times <15 hours were found mostly in the budding regions, whereas cell cycle times of 15–25 hours were mostly found in the neck region, and >25 hours were observed in the body (Fig. 7a,b). There was also a location dependence in the cell motility speed, with cells in budding and neck region having a significantly higher speed than cells in the body region (Fig. 7c). Regardless of cell location, a significant correlation was noted between cell movement speed and cell cycle duration (Fig. 7d).

**Figure 8:**
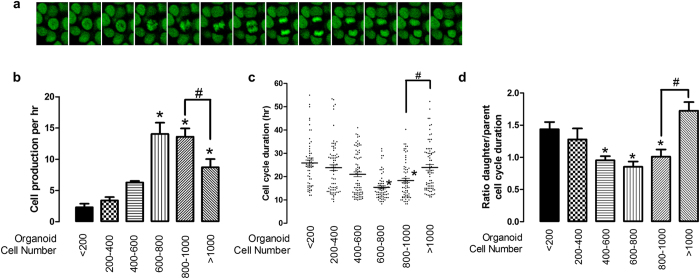
Tracking daughter cells from parent cells in taste bud organoids. Taste bud organoids were created from H2B-EGFP transgenic mice and growth monitored on the confocal microscope. **(a)** Representative image of cell division of the taste bud organoid. **(b**) H2B-EGFP was counted in 4D using Imaris software, and calculated rate of cell number changes over time (cell production/hr) from size grouping of organoids as a <200, 200–400, 400–600, 600–800, 800–1000, and >1000. **(c)** Time between cell divisions (431 cells) was tracked from 11 different taste bud organoids. Cell cycle duration was separated based on cell numbers in the organoid. **(d)** Tracking daughter cell cycle duration from parent cell (raw data shown in Supplementary Fig. 8a), and data shows the ratio of daughter cell to parent cell duration based on cell numbers in the organoid. *p < 0.05 vs. <200. ^#^p < 0.05 vs. 800–1000.

Since FUCCI2 fluorescence disappears between M (mVenus-hGem) and G1 phase (mCherry-hCdt1), it is not possible to track daughter cells. Therefore, we created taste bud organoids from H2B-EGFP transgenic mouse CV tissue to visualize cell division. H2B-EGFP organoids also confirmed that taste bud organoids display multiple layers (Supplementary Fig. 5b). In 4D live imaging, cell division clearly occurred only in the outer layer of the organoid (Fig. 8a and Supplementary video 6a and 6b). Cell numbers derived from counts of H2B-EGFP increased in a time-dependent manner, and the rate was dependent on cell number (Fig. 8b). We further determined the cell cycle duration by measuring time between cell division. In Fig. 8c, cell cycle duration was widely distributed when the organoid cell number was low, and this distribution narrowed when organoids reached 600–800 cells in size, followed by increasing distribution correlating with growth. These data complement Fig. 8b showing that the rate of proliferation reached a maximum when cell number (or organoid size) reached 600–800 cells, and the rate was significantly reduced when cell number reached >1000.

We further tracked the destiny of daughter cells following parent cell division. The production of daughter cells that had shorter cell cycle durations was increased in organoids in a size dependent manner that reached a maximum when the organoid size was 600–800 cells (Fig. 8d and Supplementary Fig. 8). In contrast, parental cells started to increase the production of a longer cell cycle in daughter cells when the organoid cell number reached 600-800 (Fig. 8d and Supplementary Fig. 8), reinforcing the idea that spatial and cell confluence feedback mechanisms impact progenitor cell growth.

## Discussion

Herein, we developed a self-renewing primary 3D-culture of CV tissue which forms organoid structures with differentiated taste cells. Recently, Yee *et al.* demonstrated by lineage tracing using Lgr5-EGFP-IRES-creERT2/Rosa26-tdTomato mice that Lgr5^+^ cells are expressed at the bottom of taste bud and trench areas at the base of the CV and give rise to differentiated taste cells^11^. More recently, it was demonstrated that flow-sorted Lgr5-GFP single cells form taste bud organoids that contain mature taste cells^19^. Similar observations in other tissues demonstrate that differentiated mature cells originate from Lgr5^+^ stem cells^13^,^15^,^17^,^18^.

In organoid culture, Wnt3a/R-spondin signaling plays an important role for maintenance of stem cells, although Wnt3a is not necessary for small intestinal organoid culture because Paneth cells provide a sustained source of Wnt signal^26^. We showed in this study that Wnt3a facilitates budding and maintenance of stem cells, since organoids grew into small spherical shapes that lacked budding under Wnt3a removal conditions. In this Wnt3a removal condition, we failed to detect T1R2 and SNAP25 mRNA in the taste bud organoid. In the CV taste buds, it is reported that β-catenin signaling is involved in taste cell fate decisions^36^. Our data suggest that Wnt3a might play an important role in stem cell regulation to differentiate T1R2 or SNAP25 in the taste bud organoids. Further studies will be needed to elucidate full differentiation pathways for each type of taste cell. We compared the expression of taste cell related genes in GI tissue derived organoids that have Lgr5^+^ cells^26^,^37^. We failed to detect T1R2 mRNA in the gastric organoid, which is consistent with other findings in the stomach^38^. However, we failed to detect T1R2 mRNA in the small intestinal organoid despite its expression in small intestinal tissue^38^. In the organoid system, it is possible to change medium conditions to induce differentiation. In fact, recent studies showed that differentiation to L-cells in the small intestinal organoid, that secretes glucagon-like peptide1 (GLP-1), is facilitated by application of NOTCH signaling inhibitor or applying short-chain fatty acids^14^,^39^. Thus, further work is needed to optimize conditions to facilitate differentiation to taste cells in GI organoids.

The Lgr5^+^ stem cell appears to play an important role in taste bud development and epithelial maintenance. Lgr5^+^ cells were competent to drive formation of differentiated taste bud organoids^19^. Other than Lgr5^+^ stem cells, the stem/progenitor pool is not well described in CV tissue. The taste bud organoid forms multiple layers representing an outer stem/progenitor compartment comprising the proliferating cells and an inner differentiated cell compartment. Using EdU staining, FUCCI2 and H2B-EGFP live imaging, we were able to demonstrate the existence of these 2 compartments. In the outer cell layer of organoids, we found a CD44 and SOX9 positive cell population. Both CD44 and SOX9 are widely recognized as stem/progenitors in several tissues^28^,^30^,^31^,^40^,^41^, although it is still unclear what type of stem/progenitor cells express these genes. CD44 seems to widely mark stem/progenitor cells, including Lgr5^+^ cells in the small intestine^30^,^31^. We detected high expression of Lgr5 mRNA in CD44^+^ sorted cells and successfully generated taste bud organoids from CD44^+^ sorted cells, suggesting that CD44 also marks stem/progenitor cells. Additionally, it has also been demonstrated that CD44 plays an important role in controlling cell-cell interaction, cell adhesion, proliferation as well as cell migration^42^,^43^,^44^,^45^. We observed that proliferating cells move in the outer layer of taste bud organoids where CD44 is present, suggesting that CD44 may contribute to cell migration in the stem/progenitor niches.

We further visualized how taste bud organoids grow from YC, FUCCI2 or H2B-EGFP mouse derived CV tissue. Surprisingly, proliferating cells move within the outer layer of taste bud organoids while the non-proliferative taste cells remain relatively fixed within the inner cell layers. By utilizing H2B-EGFP reporters, we found several distinct cell cycles in smaller organoids, which changed to a more uniform 12-hour cycle when organoids increased in size to 600–800 cells. At this time, we observed the increase of T1Rs mRNA in the taste bud organoid (DAY5 in Fig. 1d). In contrast, in the initially generated organoid (<400 cells), many stem/progenitor cell cycles lasted approximately 24 hours, and those cells frequently generated shorter cell cycle daughter cells. About the same size with FUCCI2 organoids, we observed robust 24-hour mVenus-hGem (proliferative cell) oscillation, consistent with *in vivo* findings that showed one peak of proliferation activity during 24 hours. This suggests that the longer cycle cells are stem cells while the shorter cycle cells function as progenitor cells. Furthermore, the shorter cell cycle cells were mostly found in the budding area engaged in movement while the longer cycle cells were seen in the sphere body of the organoids. Sullivan *et al.* found rapid cycling progenitors-like cells in the basal area surrounding (perigemmal) the taste bud in CV tissue, while a few % of slow cycling stem-like cells were also present in the basal compartment of the taste bud^10^. It was also speculated that Lgr5^low^ cells function as progenitors in the base of taste buds while Lgr5^high^ cells at the trench act as a stem cell pool that gives rise to Lgr5^low^ progenitors^32^. Since this area consists of CD44 positive cells, we are in agreement with Feng *et al.* that 1) stem cells at the bottom of the trench create progenitor cells, which subsequently migrate to the base of the taste bud, and 2) stem cells at the bottom of the taste bud generate progenitor cells^32^. Additionally, since slow cell cycle cells have motility in the organoid, we further speculate that 3) the stem cell itself migrates from the bottom of the trench to the base of the taste bud, followed by generation of fast cell cycle progenitor cells. Further stem/progenitor tracing experiments are needed in the organoid system as well as *in vivo*.

The role of individual stem/progenitor cells that have a different cell cycle duration is still unknown, especially the longer cell cycle cells^46^. It has been reported that cell cycle is tightly coupled with circadian clock genes^47^. Notably, although the master pacemaker of circadian rhythms resides in the suprachiasmatic nucleus within the hypothalamus, circadian rhythms exist even in single cells in peripheral tissues^48^. We previously reported autonomously synchronized circadian rhythms in the small intestinal organoids^49^. Interestingly, circadian clocks in gustatory receptor neurons generate taste sensitivity rhythm and the peak appears in the morning in the Drosophila taste organ^50^, similar to the 24 hour proliferation activity rhythm, with a peak in the morning, observed in the mouse tongue^10^. During regulation of the circadian rhythm by clock genes, some clock proteins, such as PERIOD, are reported to extend cell cycle duration via activation of *p16* (*Ink4A*), resulting in inhibition of G1-S transition^51^. Our data demonstrates that cell cycle duration is dependent on the G1 phase, therefore a longer cell cycle may be generated by regulation of clock genes to control proliferative activity within the stem/progenitor cells. On the other hand, it is reported that the amplitude of clock gene oscillation is reduced in the late phase of primary cultures due to the lack of synchronization^52^. This could support our observation that the major 24-hour synchronized oscillation of proliferation activity disappeared in the late phase of FUCCI2 organoid growth.

We identified two types of organoids, 1) spheres with small budding and 2) spheres with prominent budding while maintaining a spherical structure. The different rates of proliferation observed between types may represent organoids derived from different populations of Lgr5^+^ stem cells. The presence of fast versus slow cycling stem populations alludes to separate stem/progenitor populations *in vivo*^10^, but the identification of these populations and functional consequences have yet to be determined.

We have shown that taste bud organoids form multiple layers, i.e., an outer stem/progenitor compartment and an inner differentiated cell compartment that mimics the architecture of CV tissue. In contrast, the mature taste bud in the native tissue has a single taste pore where the microvilli of taste cells project. We failed to find a taste pore in the taste bud organoid although we observed several lumens to be present within the organoids. Consistent with previous findings, the distribution of taste cells were heterogeneous in the organoids^19^. It is reported that taste nerve innervation as well as Sonic hedgehog (Shh) play important roles in both taste cell development and taste pore formation^53^,^54^,^55^,^56^, whereas our current growth conditions were sufficient to induce taste cell differentiation^19^. Application of Shh, however, did not affect taste cell differentiation in the taste bud organoid culture^19^, therefore addition of neuronal factors or co-culture with taste neuron may facilitate taste cell differentiation and control the formation of the architecture (including a single taste bud pore) that is found in the taste bud *in vivo*.

The presence of differentiated taste cells within organoids should also provide a novel platform for studying taste function and responses, and furthermore may be useful in studying drug applications directed at altering taste cell function. We found that stem/progenitor markers were expressed in the CV tissue as well as in the organoids. Tissue and stem cell-derived taste bud organoids express mature taste cells whose differentiation is dependent upon Wnt signaling, however it will be interesting to use this platform to study the pathways that direct taste bud regeneration following injury, differentiation and the impact of circadian rhythm on the development of specific taste cell subtypes, and factors that regulate their function.

## Material and Methods

### Animals

Experiments used C57BL/6J (Jackson Laboratory), yellow cameleon 3.0 transgenic^25^, Lgr5-EGFP-IRES-creERT2 (Jackson Laboratory), Gt(ROSA)26Sortm4(ACTB-tdTomato,-EGFP)Luo/J (Jackson Laboratory), R26-H2B-EGFP (Riken Acc. No. CDB0238K)^35^ or R26p- FUCCI2 (Riken Acc. No. CDB0203T)^24^ mice. Mice were maintained in an AAALAC approved facility and all animal studies followed protocol 04-03-08-01 that was approved by the Institutional Animal Care and Use Committee of the University of Cincinnati. All experiments were carried out in accordance with the approved ethical guidelines and regulations.

### Organoid culture

Tongue was isolated and dispase II (Roche, 1 mg/mL) was injected under the epithelium. After 30 min incubation at room temperature, the epithelium was peeled away under the dissecting scope, and then CV tissue was isolated. The CV tissue was incubated with 0.25% trypsin/EDTA for 30 min at 37 °C and centrifuged at 800 g for 5 min. The tissue was suspended in Matrigel (BD biosciences). Gastroids (fundus and antrum) and enteroids were generated as described before^26^. Briefly, isolated stomach or small intestine were incubated at 4 °C under agitation respectively for 2 hrs or 30 min in DPBS (w/o Ca^2+^/Mg^2+^) with 5 or 2 mM EDTA (Sigma). Then, tissue was placed into 5 ml cold dissociation buffer (43.4 mM sucrose, 54.9 mM D-sorbitol, in DPBS), and shaken forcefully for 2 min to dissociate individual glands or crypts from tissue. Dissociated glands or crypts were centrifuged at 150 x*g* for 5 min at 4 °C, and the pellet re-suspended in Matrigel.

The suspended glands or crypts were seeded into 12-well culture plates (50 μl Matrigel). After Matrigel polymerization at 37 °C, advanced DMEM/F12 supplemented with 2 mM GlutaMax, 10 mM HEPES, 100 U/mL penicillin/100 μg/mL streptomycin, 1 × N2 and 1 × B27 supplements (Life Tech) plus the following growth factors was added to the wells and replaced every 4 days; **Taste bud organoid medium:** Wnt-conditioned media (50%), R-spondin-conditioned media (10%), EGF (50 ng/mL, Pepro Tech), Noggin (100 ng/mL, Pepro Tech). **Enteroid:** R-spondin-conditioned media (10%), EGF (50 ng/mL), Noggin (100 ng/mL). **Gastroid:** Wnt-conditioned media (50%), R-spondin-conditioned media (10%), [Leu15]-Gastrin I (10 nM, Sigma), nAcetylcysteine (1mM: Sigma), FGF10 (100 ng/mL, Pepro Tech), EGF (50 ng/mL), Noggin (100 ng/mL).

### Organoid passage

Organoids in Matrigel were collected with cold DPBS (w/o Ca^2+^/Mg^2+^) and centrifuged at 150 × *g* for 5 min at 4 °C, followed by removing supernatants including Matrigel. Taste bud organoids were incubated with 0.25% trypsin/EDTA for 30 min at 37 °C, and then dissociated into single cells through a 31G insulin needle. After centrifuged at 800 x*g* for 5 min, cells were re-suspended with Matrigel. Gastrointestinal organoids were passaged, according to as previously described^26^. All experiments including immunostaining, PCR or live imaging were conducted in organoids after 2–3 passages.

### Cell Sorting

CV tissue, isolated from Lgr5-EGFP mice, was dissociated with 1 mg/mL collagenase A (Roche) and 2 mg/mL dispase II for 30 min at 37 °C followed by incubation with 0.25%trypsin-EDTA for 30 min at 37 °C . Organoids were dissociated with TrypLE™ express (Invitrogen) supplemented with 10 μM Y-27632 for 4 min at 37 °C. Dissociated cells were then pushed through an insulin syringe. Cell clumps were removed using 35 μm cell strainer (Fisher Scientific) and the flow-through was pelleted at 500 x*g* at 4 °C for 5 min. Single cells pellets were resuspended in sorting buffer (5% BSA, 10 mM HEPES, 0.5 mM EDTA in DPBS). Cells were stained with PE-Cy7-conjugated CD44 antibody (Biolegend) and incubated for 30 min on ice. Cells were then washed and resuspended in sorting buffer. 7-AAD (eBiosciences) was added 20 min prior FACS-sorting. A FACSAria II equipped with a 100 μm nozzle was used (BD Biosciences). Cells were sorted into 500 μL sorting buffer for single cell culture. Sorted cells were collected, pelleted, and embedded in Matrigel.

### Live imaging

Organoids were grown in an 8-well Lab-Tek chamber with coverglass (Thermo Scientific). Imaging was performed in organoid culture medium under 5% CO_2_/37 °C (incubation chamber, PeCon, Erbach, Germany) on an inverted confocal microscope (Zeiss LSM710). Organoid growth was monitored using a Zeiss Plan-Apochromat x20 objective. YFP (Ex: 514 nm, Em: 535–685 nm), H2B-EGFP (Ex: 488 nm, Em: 500–550 nm) and FUCCI2 (mCherry-hCdt1(30/120), Ex: 560 nm, Em: 580-630 nm, and mVenus-hGem(1/110), Ex: 514 nm, Em: 520-550 nm) were monitored at 10 to 30 min intervals. At each time point, a z-stack was taken at 3–5 μm focus intervals. The 4D movies or 3D images were rendered by Imaris 7.7 (Bitplane) or Volume Rendering Program (Voxx 2.15, Indiana University), respectively. The 2D images were processed by Zen 2012 software (Zeiss).

### Immunostaining

Mouse tongue and organoids were fixed in 4% paraformaldehyde for 30 min, followed by OTC embedding and freezing. Section was stained with hematoxylin & eosin. Images were taken using the Nikon ECLIPSE TE 200-U microscope (Camera: Qimage digital camera, software: Qcapture pro). Sections (10 μm) for immunofluorescence were blocked with 3% BSA for 1 hr. Sections were then incubated for 1 hour at room temperature with first or secondary antibodies listed below. Nuclear stain (Hoechst 33342, Ex: 405, Em: 420–470 nm, 1 μg/ml, Invitrogen) for 1 min was also performed.

Whole mount staining was performed on organoids. Organoids resuspended in Matrigel were fixed with 4% paraformaldehyde for 30 min at room temperature, followed by tissue permeabilization with 0.1% Triton X-100 in PBS for 20 min, then blocked in 5% BSA for 1 hr. Organoids were incubated with first or secondary antibodies listed below overnight at 4 °C, followed by nuclear stain (Hoechst 33342, 10 μg/ml, Invitrogen) for 20 min. Whole mount sections were obtained via z-stack reconstruction using the Zeiss LSM710.

The following pairs of first and secondary antibodies were used: anti-GFP (rabbit polyclonal, 1:200, Abcam) and Alexa Fluor 488 goat anti-rabbit IgG (1:400, Invitrogen); anti-T1R3 (rabbit polyclonal, 1:200)^57^, anti-Gustducin (rabbit polyclonal, 1:200, Santa Cruz), or anti-SOX9 (rabbit polyclonal, 1:200, Millipore), and Alexa Fluor 647 donkey anti-rabbit IgG (1:400, Invitrogen); anti-CD44 (rat monoclonal: 1 : 200, Abcam or Alexa 647-conjugated anti-CD44, Biolegend) or anti-E cadherin (rat monoclonal: 1 : 200, Santa Cruz) and Alexa Fluor 488 goat anti-rat IgG (1:400, Invitrogen) or UEA1 (Rhodamin-conjugated, Ex: 560 nm, Em: 570–620 nm, 1:400 for 1 hr, Vector). Protein labeled with Alexa fluor 488 was imaged at 500-550 nm in response to 488 nm excitation, while Alexa fluor 647 was imaged at 650–700 nm in response to 633 nm excitation in the Zeiss LSM710 confocal microscope.

EdU staining to analyze cell proliferation was performed using the Click-iT EdU Alexa Fluor 594 kit (Invitrogen). Organoids were incubated with 5 μM EdU for 1 hr followed by fixation for 15 min with 3.7% formaldehyde. The Click-iT reaction cocktail was added according to manufacturer’s protocol and incubated for 30 min. Nuclear stain (Hoechst 33342, 10 μg/ml) was added for 20 min. Whole mount images were obtained via z-stack reconstruction using the Zeiss LSM710.

### Transmission Electron Microscopy

Organoids were harvested and fixed in 3% glutaraldehyde/0.175M cacodylate buffer over night at 4 °C. The fixed organoids were postfixed in 1% osmium tetroxide/0.175M cacodeylate buffer, then processed and embedded in LX-112 resin. Thin sections were stained with uranyl acetate and lead citrate, and analyzed with Hitachi H7600 transmission electron microscope.

### Detection of mRNA

Total RNA was isolated from CV tissue or cultured organoids. cDNA was synthesized (High Capacity cDNA Reverse Transcription Kit, Applied Biosystems). cDNA was amplified by real-time PCR (TaqMan, Applied Biosystems) or regular PCR (FastStart PCR, Roche), using the following primers: CD44 (Mm01277163_m1, TaqMan primer, Invitrogen), GAPDH (Forward: 5′-AACGACCCCTTCATTGAC-3′ and Reverse: 5′-TCCACGACATACTCAGCAC-3′), Lgr5 (Forward: 5′-CCTACTCGAAGACTTACCCAGT-3′ and Reverse: 5′-GCATTGGGGTGAATGATAGC-3′), Sox9 (Forward: 5′-CGGAACAGACTCACATCTCTCC-3′ and Reverse: 5′-GCTTGCACGTCGGTTTTGG-3′), Gustducin (Forward: 5′-TCATCCATAAGAATGGTTACAGC-3′ and Reverse: 5′-CCCACAGTCGTTTAATGATTTC-3′), T1R1 (Forward: 5′-TTCCTTGGTAGCTGGGAGTTGC-3′ and Reverse: 5′-TCAGGTAGTGCCGCAGCGCCTC-3′), T1R2 (Forward: 5′-ATGAAGGTCTTGGGCTACAAC-3′ and 5′-CTGGAAGGCAATGCAGATATCG-3′), T1R3 (Forward: 5′-GTTGCAGAACTTCAGCTGGAAC-3′ and Reverse: 5′-TCATGACCAGGTCAGATGTCAG-3′), NTPDase2 (Forward: 5′-GACAAGGAAAATGACACAGGTATCGTGG-3′ and Reverse: 5′-GTTCAAGACATTCAACCAGACTC-3′)^58^, SNAP25 (Forward: 5′-TGCTGCAGCTGGTTGAAGAGAGTA-3′ and Reverse: 5′-ACTTCCCAGCATCTTTGTTGCACG-3′), TRPV1 (Forward: 5′-CTGTCCAGGAAGTTCACTGAATG-3′ and Reverse: 5′-CTAGTAGAAGATGCGCTTGAC-3′), TRPM5 (Forward: 5′-GTCTGGAATCACAGGCCAAC-3′ and Reverse: 5′-GTTGATGTGCCCCAAAAACT-3′), PLCβ (Forward: 5′-GCCAGTTCTCAGGCCTTTCCTC-3′ and Reverse: 5′-TCTTCTACAGGGACACTAGACG-3′), CaSR (Forward: 5′-TGCCAAGGAGATTGAGTTCC-3′ and Reverse: 5′-GTAGGACAGCTCTCGGTTGG-3′).

### Cell cycle analysis

The number of nuclei or cell cycle duration of FUCCI2- and H2B-EGFP- taste bud organoids were tracked by Imaris 7.7 automatically or manually, respectively. The multivariate Gaussian distribution equation for the fitting of distributions of cell cycle duration in taste bud organoids are given by




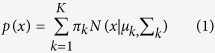




where μ is mean and Σ is variance (Supplementary Fig. 6). Regression analysis, except the distribution of cell cycle duration, was done with power function (Fig. 4c,d and Fig. 7d). Curve fitting of the data was performed with Igor Pro (WaveMetrics) software.

Fast Fourier transform (FFT) analysis for the time course changes of number of mVenus-hGeminin positive cells (normalized by the peak value) was performed with the program written by Uhlen^59^ (Fig. 5).

### Statistical analysis

All values are reported from representative experiments as the mean ± standard error of the mean (SEM) from multiple experiments. Statistical significance was determined using unpaired Student’s T-test (in Figs 5d^#^, 7b^#^, 8b–d^#^), or one-way ANOVA with Dunnett’s multiple comparison post-hoc test (in Figs 5d*,h, 7b*,c*, 8b–d*). A p value of <0.05 was considered significant.

## Additional Information

**How to cite this article**: Aihara, E. *et al.* Characterization of stem/progenitor cell cycle using murine circumvallate papilla taste bud organoid. *Sci. Rep.* **5**, 17185; doi: 10.1038/srep17185 (2015).

## Supplementary Material

Supplementary Video 1

Supplementary Video 2

Supplementary Video 3a

Supplementary Video 3b

Supplementary Video 4a

Supplementary Video 4b

Supplementary Video 5

Supplementary Video 6a

Supplementary Video 6b

Supplementary Information
