# Supplementary material for: Characterization of stem/progenitor cell cycle using murine circumvallate papilla taste bud organoid
Source: Sci Rep. 2015 Nov 24;5:17185. doi: 10.1038/srep17185 (PMC4665766; doi:10.1038/srep17185)
Supplement: Supplementary Information [file srep17185-s10.pdf]

Characterization of stem/progenitor cell cycle using murine circumvallate papilla taste bud organoid

Eitaro Aihara[1*], Maxime M. Mahe[2], Michael A. Schumacher[1], Andrea L. Matthis[1], Rui Feng[1], Wenwen Ren[3], Taeko K. Noah[4], Toru Matsu-ura[1], Sean R. Moore[4], Christian I. Hong[1], Yana Zavros[1], Scott Herness[5], Noah F. Shroyer[4], Ken Iwatsuki[6], Peihua Jiang[3], Michael A. Helmrath[2] and Marshall H. Montrose[1]

Affiliation:

1. Department of Molecular and Cellular Physiology, University of Cincinnati, Cincinnati, Ohio
2. Division of Pediatric Surgery, Cincinnati Children's Hospital Medical Research Center, Cincinnati, Ohio
3. Monell Chemical Senses Center, Philadelphia, Pennsylvania
4. Division of Gastroenterology, Hepatology, and Nutrition, Cincinnati Children's Hospital Medical Research Center, Cincinnati, Ohio
5. Division of Oral Biology, The Ohio State University, Columbus, Ohio
6. Department of Nutritional Science and Food Safety, Tokyo University of Agriculture, Tokyo, Japan

***Correspondence to:** Eitaro Aihara

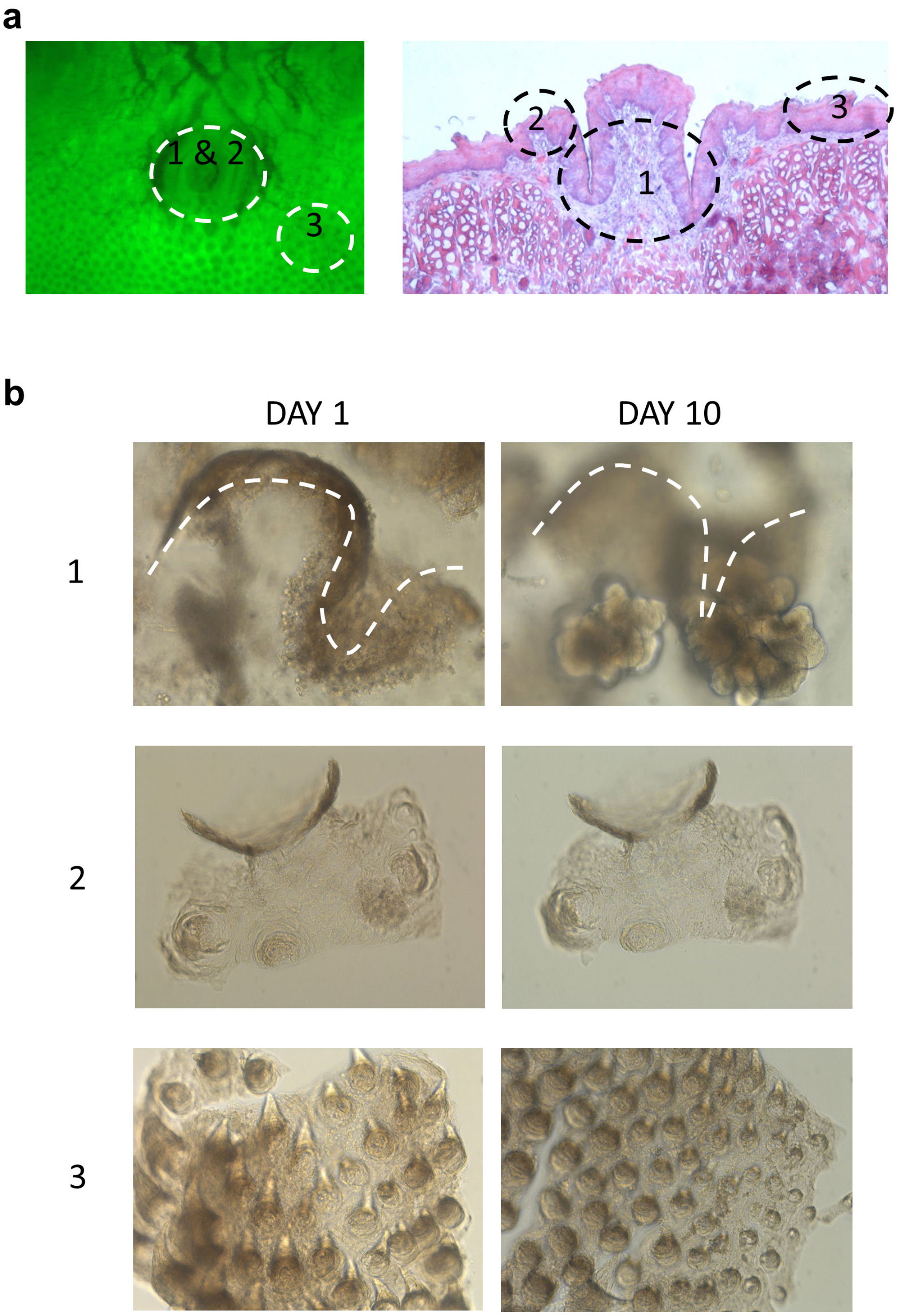

Supplementary Figure 1: Regional specificity for taste bud organoid generation. **(a)** YFP fluorescence image and H&E staining of isolated tongue from YC mouse. **(b)** Different area (1, 2, 3 correspond *dotted line* in **a**) of CV tissue was embedded in Matrigel and images were taken at Day 1 and 10. The *white dotted line* shows morphology of CV epithelium. It is noted that only region '1' generated taste bud organoids.

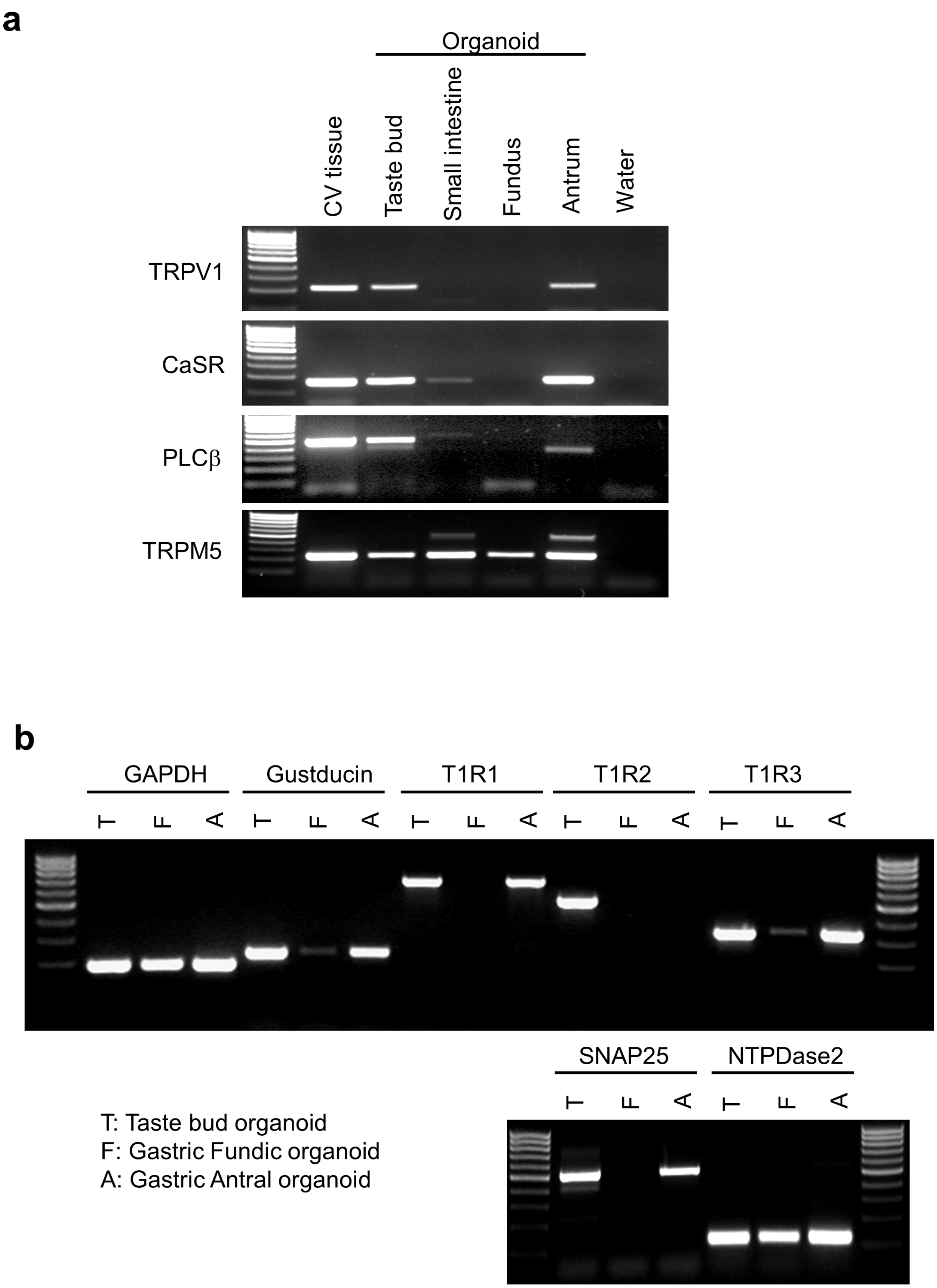

Supplementary Figure 2: Expression of taste cell lineage marker mRNA in gastrointestinal organoids compared with taste bud organoids. (a) Calcium signal-related receptor/channels, TRPV1, CaSR, PLCβ2, TRPM5 mRNA were determined in CV tissue, taste bud organoid, small intestinal orgnaoid, fundic organoid as well as antral orgnaoid. **(b)** Taste cell marker mRNA was determined in taste bud organoids, fundic organoids, and antral organoids.

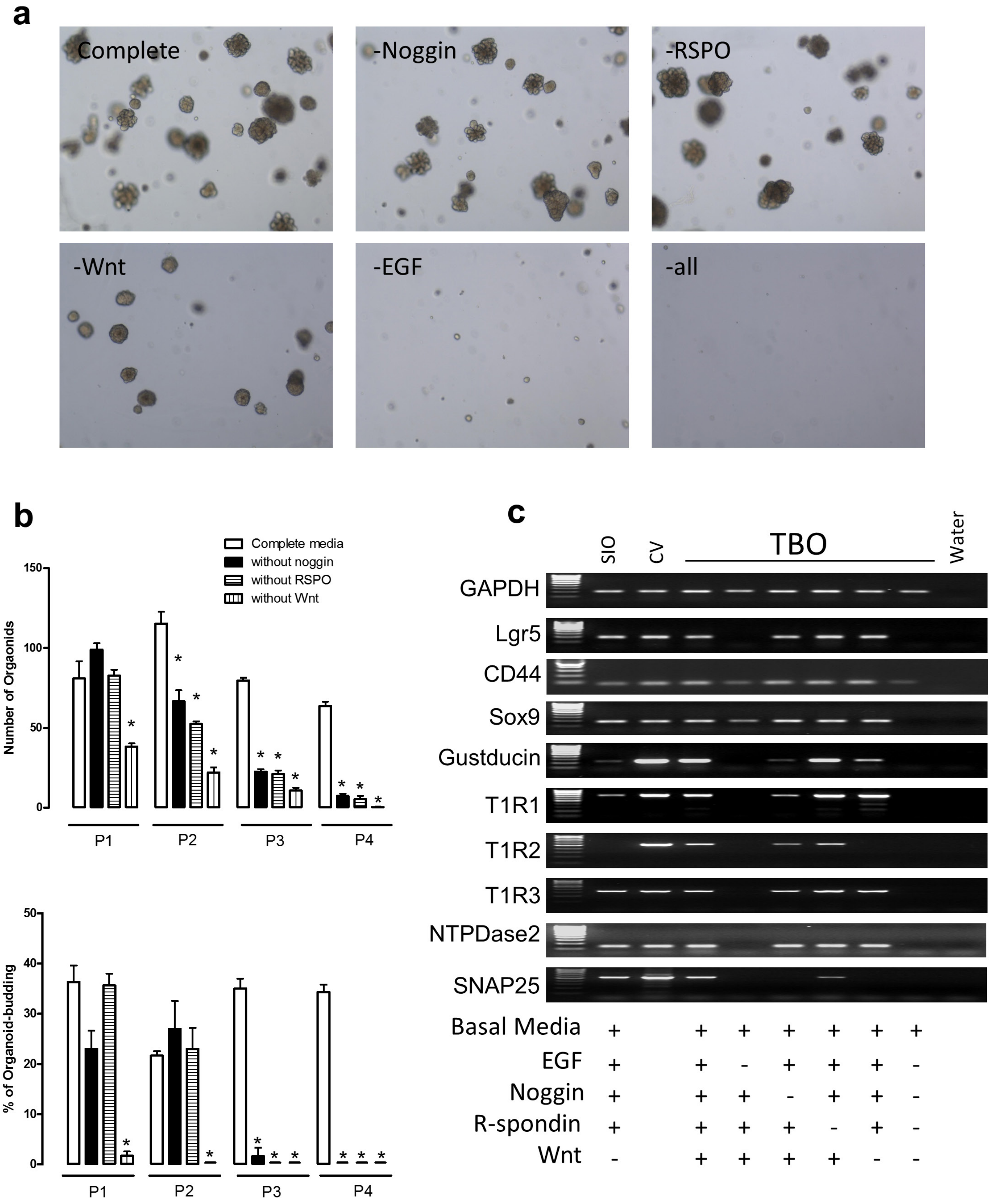

Supplementary Figure 3: Determination of optimal growth conditions for taste bud organoids. (a) After organoid passage, the indicated growth factor was removed from culture medium. Representative images were taken at 12 day of culture. **(b)** Organoids grown in each growth factor removed medium condition were counted. In addition, the percentage of budding organoids per seeded cells was analyzed. 500 cells were embedded in Matrigel on each passage. *, p<0.05 vs. complete medium. **(c)** Stem/progenitor cell or taste bud lineage maker mRNA was determined by RT-PCR on each condition. SIO: small intestinal organoid, CV: CV tissue, TBO: taste bud organoid.

a

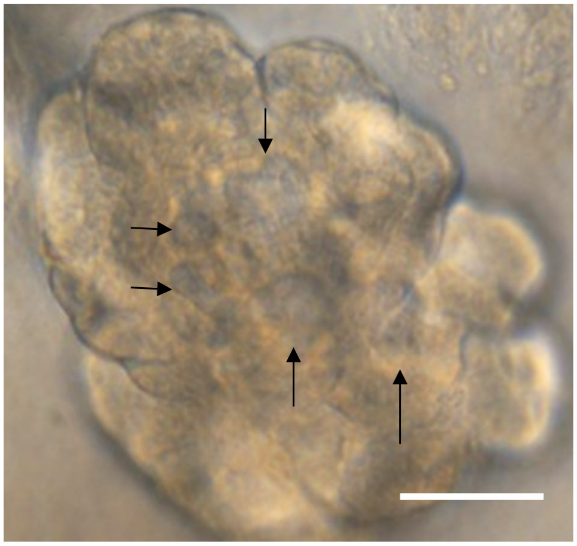

b

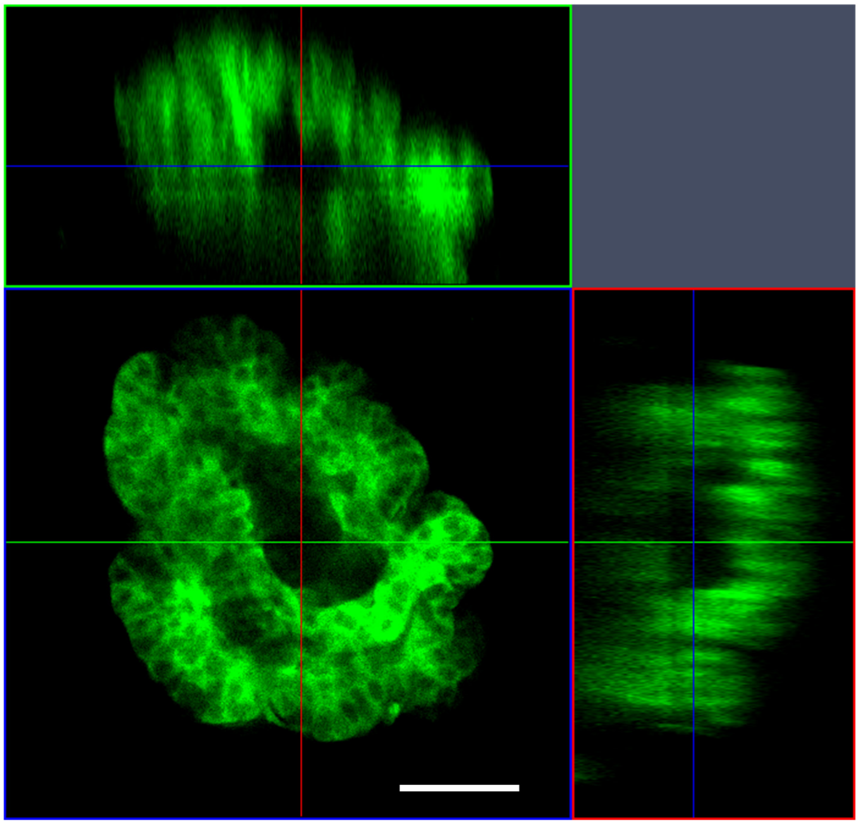

Supplementary Figure 4: The presence of lumen in the center of taste bud organoid. Taste bud organoids grown from isolated CV from YC mouse and is shown as bright field image **(a)** and confocal image **(b)**. Arrows in **a** indicate the luminal structures. Scale bars = 50 µm.

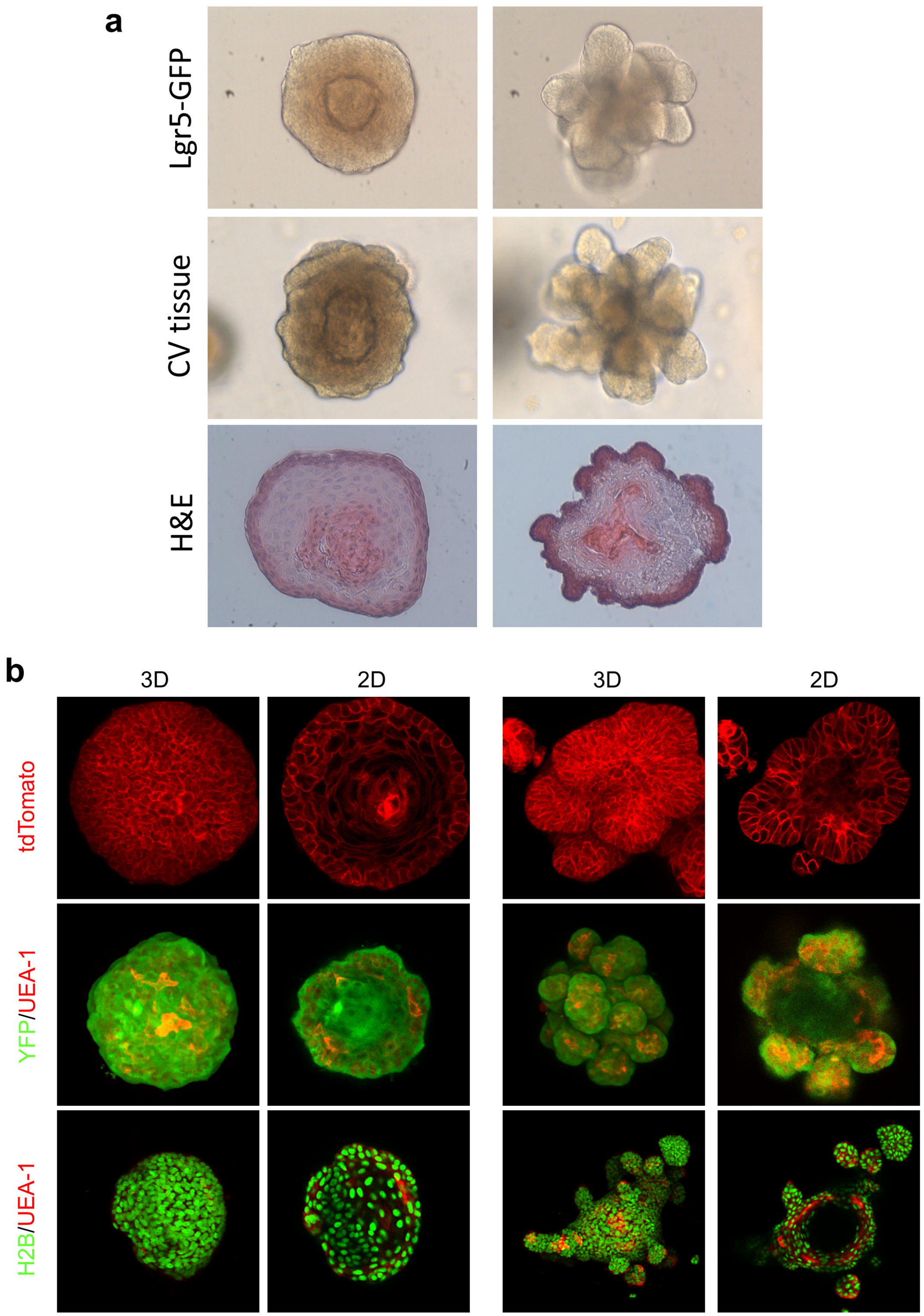

Supplementary Figure 5: Different growth appearances of taste bud organoids. (a) Taste bud organoids created from sorted Lgr5⁺-GFP cells or CV tissue of C57BL/6 mice are shown as bright field and H&E staining images. **(b)** Taste bud organoids created from membrane-tdTomato (red), YC (green) or H2B-EGFP (green) mice CV tissue. YC or H2B-EGFP taste bud organoids were stained with UEA-1.

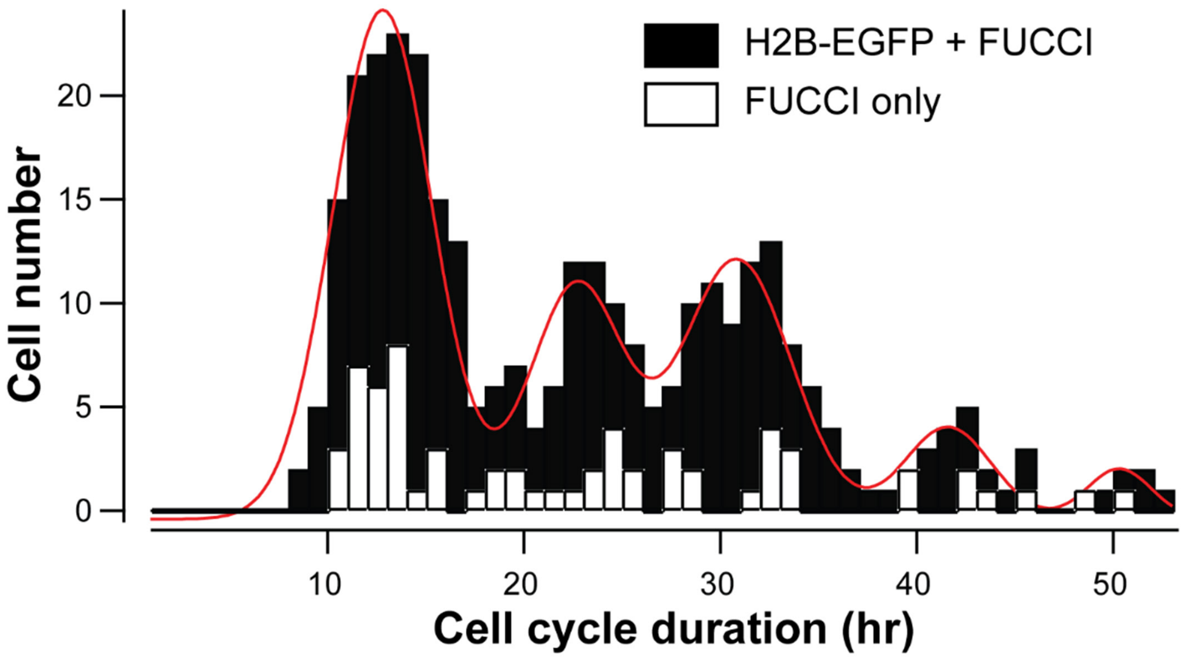

Supplementary Figure 6: Distribution of cell cycle duration in taste bud organoids. Black bars show the distribution of the sum of cell cycle duration obtained from H2B-EGFP and FUCCI2 organoids, while white bars show only the distribution of the cell cycle duration in FUCCI2 organoids. The distribution in the sum of H2B-EGFP and FUCCI2 were fitted to multivariate Gaussian distribution (red line).

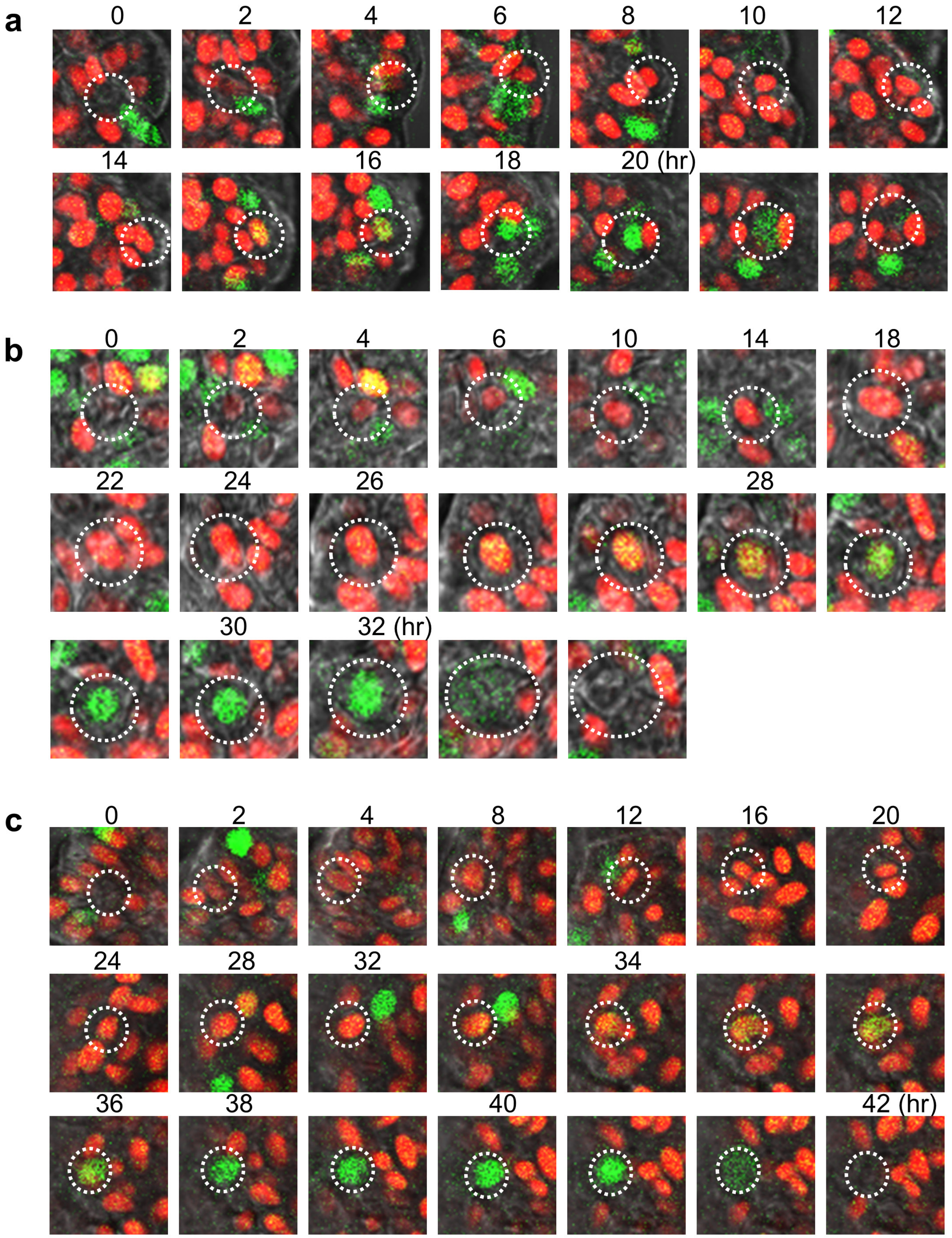

Supplementary Figure 7: Tracking cell cycle in taste bud organoids. Representative images of 22 (**a**), 34 (**b**) or 42 (**c**) hour cell cycles determined by tracking FUCCI2 fluorescence appearance in the taste bud organoids.

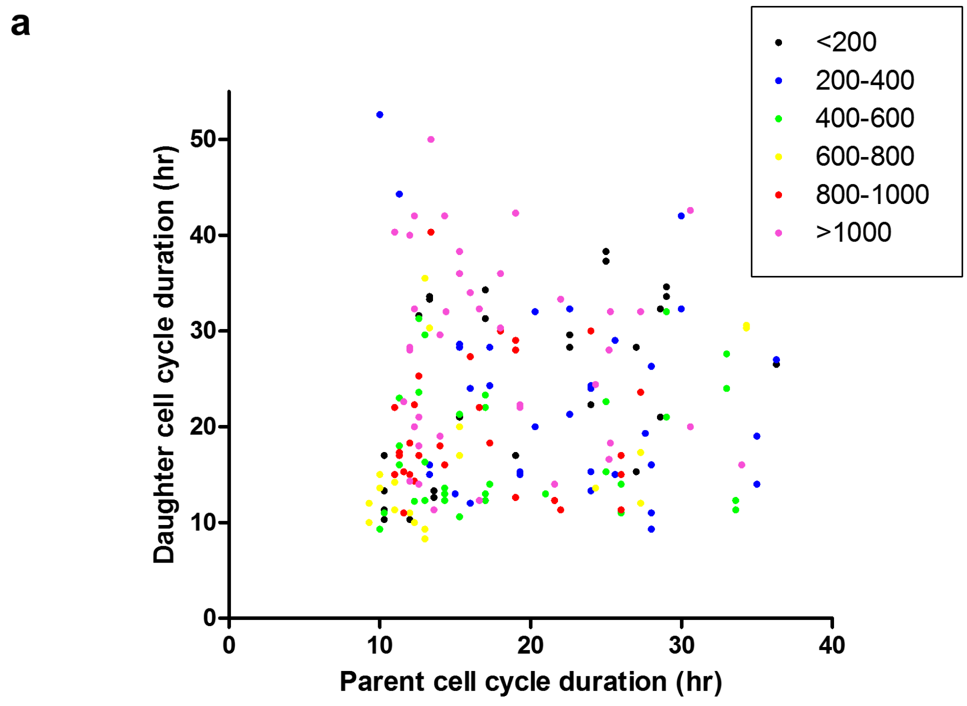

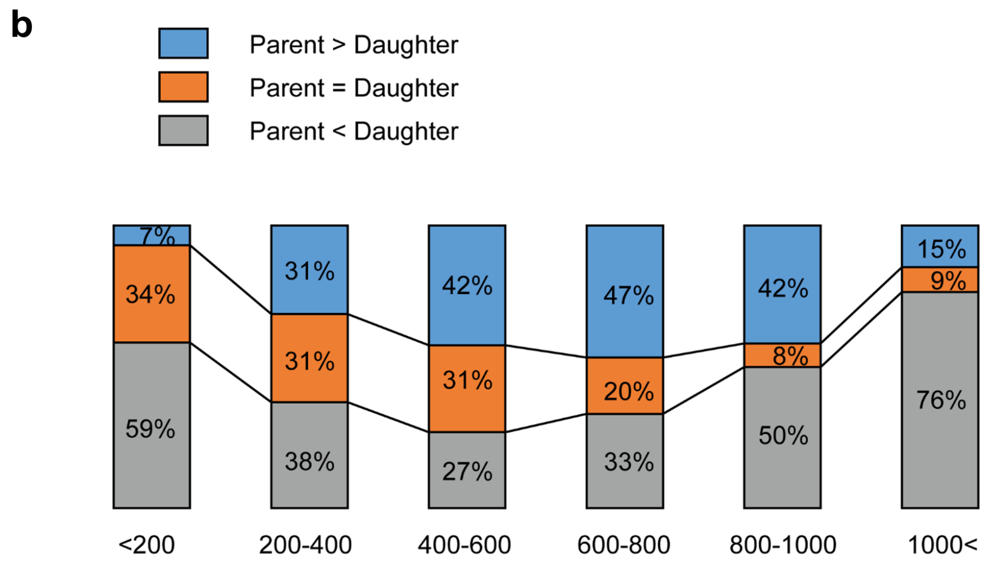

Supplementary Figure 8: Tracking daughter cells from parent cells in taste bud organoids. Taste bud organoids were created from H2B-EGFP transgenic mice and growth was monitored on the confocal microscope. **(a)** Tracking daughter cell cycle duration from parent cell, and data shows parent cell vs. daughter cell duration when cell numbers in organoid are <200 (32 tracks), 200-400 (37 tracks), 400-600 (34 tracks), 600-800 (26 tracks), 800-1000 (31 tracks), and >1000 (41 tracks). **(b)** The percentage of cell populations whose parent cell has longer, equal, or shorter cell cycle duration than its daughter cell has. The populations were defined by the ratio of cell cycle durations of the daughter cell divided by that of the parent cell (Ratio > 1.15: blue, 0.85 ≦ Ratio ≦ 1.15: orange, Ratio < 0.85: grey). A daughter to parent ratio 0.15 gives a difference of 2.85 ± 1.07 hr.

Movie Legends

Supplementary Video 1. 4D YFP organoid growth from 2-5 days. Taste bud organoid created from YC mouse. YFP image taken in 30 minute intervals with z-stack using confocal microscope and reconstructed 3D over the time using Imaris 7.7.

Supplementary Video 2. 4D YFP organoid growth from 9-12 days. Taste bud organoid created from YC mouse. YFP image taken in 30 minute intervals with z-stack using confocal microscope (Zeiss LSM710, Zeiss Plan-Apochromat x20 objective) and reconstructed 3D over the time using Imaris 7.7.

Supplementary Video 3. FUCCI2 organoid growth from 3-6 days. Taste bud organoid created from FUCCI2 transgenic mouse. FUCCI2 image taken in 40 minute intervals with z-stack using confocal microscope (Zeiss LSM710, Zeiss Plan-Apochromat x20 objective) and reconstructed in 3D (**a**) or 2D (**b**) over the time using Imaris 7.7. This is a complemental movie to **Fig. 5a**.

Supplementary Video 4. FUCCI2 organoid growth from 9-12 days. Taste bud organoid created from FUCCI2 transgenic mouse. FUCCI2 image taken in 40 minute intervals with z-stack using confocal microscope (Zeiss LSM710, Zeiss Plan-Apochromat x20 objective) and reconstructed in 3D (**a**) or 2D (**b**) over the time using Imaris 7.7. This is a complemental movie to **Fig. 5e**.

Supplementary Video 5. Tracking individual cells in taste bud organoid. Proliferative cells were traced by Imaris 7.7 on FUCCI2 organoid showed in **Fig. 5e** and **Supplementary video 4**. This is a complemental movie to **Fig. 7a**.

Supplementary Video 6. H2B-EGFP organoid growth. Taste bud organoid created from H2B-EGFP transgenic mouse. GFP image taken in 5 minute intervals with z-stack using confocal microscope (Zeiss LSM710, Zeiss Plan-Apochromat x20 objective) for 15 hours and reconstructed in 3D (**a**) or 2D (**b**) over the time using Imaris 7.7.
